# Wild ducks excrete highly pathogenic avian influenza virus H5N8 (2014–2015) without clinical or pathological evidence of disease

**DOI:** 10.1038/s41426-018-0070-9

**Published:** 2018-04-18

**Authors:** Judith M. A. van den Brand, Josanne H. Verhagen, Edwin J. B. Veldhuis Kroeze, Marco W. G. van de Bildt, Rogier Bodewes, Sander Herfst, Mathilde Richard, Pascal Lexmond, Theo M. Bestebroer, Ron A. M. Fouchier, Thijs Kuiken

**Affiliations:** 1 Department of Viroscience, Erasmus MC, P.O. Box 2040, 3000 CA Rotterdam, The Netherlands; 2 Department of Pathobiology, Faculty of Veterinary Medicine, Utrecht University, Postbus 80163, 3508 TD Utrecht, The Netherlands; 3 Department of Biology and Environmental Sciences, Faculty of Health and Life Sciences, Linnaeus University, 391 82 Kalmar, Sweden; 4 Department of Farm Animal Health, Faculty of Veterinary Medicine, Utrecht University, Postbus 80163, 3508 TD Utrecht, The Netherlands

## Abstract

Highly pathogenic avian influenza (HPAI) is essentially a poultry disease. Wild birds have traditionally not been involved in its spread, but the epidemiology of HPAI has changed in recent years. After its emergence in southeastern Asia in 1996, H5 HPAI virus of the Goose/Guangdong lineage has evolved into several sub-lineages, some of which have spread over thousands of kilometers via long-distance migration of wild waterbirds. In order to determine whether the virus is adapting to wild waterbirds, we experimentally inoculated the HPAI H5N8 virus clade 2.3.4.4 group A from 2014 into four key waterbird species—Eurasian wigeon (*Anas penelope*), common teal (*Anas crecca*), mallard (*Anas platyrhynchos*), and common pochard (*Aythya ferina*)—and compared virus excretion and disease severity with historical data of the HPAI H5N1 virus infection from 2005 in the same four species. Our results showed that excretion was highest in Eurasian wigeons for the 2014 virus, whereas excretion was highest in common pochards and mallards for the 2005 virus. The 2014 virus infection was subclinical in all four waterbird species, while the 2005 virus caused clinical disease and pathological changes in over 50% of the common pochards. In chickens, the 2014 virus infection caused systemic disease and high mortality, similar to the 2005 virus. In conclusion, the evidence was strongest for Eurasian wigeons as long-distance vectors for HPAI H5N8 virus from 2014. The implications of the switch in species-specific virus excretion and decreased disease severity may be that the HPAI H5 virus more easily spreads in the wild-waterbird population.

## Introduction

Avian influenza viruses—in particular highly pathogenic avian influenza (HPAI) viruses—form a continuous threat to the poultry industry, public health, and to some wild bird species. Since the emergence of the HPAI H5N1 virus in the poultry in China in 1996, H5 HPAI viruses that share a common ancestral virus (A/goose/Guangdong/1/96 [GsGd]) have continued to cause outbreaks in poultry^1^. These outbreaks were associated with the first human infections caused by the HPAI H5N1 virus and with the spillover of HPAI H5N1 virus to wild birds. The hemagglutinin (H) gene of the HPAI H5N1 virus diversified into multiple genetic lineages (“clades”); more recently, reassortment between the HPAI H5N1 virus and the low pathogenic avian influenza (LPAI) viruses resulted in HPAI viruses with neuraminidase (N) genes (N1, N2, N5, N6, and N8) and other genes of LPAI virus origin^2–5^. From China, H5 GsGd virus has been introduced to other Asian countries, the Middle East, Africa, and Europe. Within Europe, HPAI H5N1 GsGd virus has been detected in multiple countries in 2004 (clade 1), 2005/2006/2007 (clades 2.2 and 2.2.1), and 2008/2009/2010 (clade 2.3.2)^6, 7^. In November and December of 2014, the HPAI H5N8 GsGd virus (clade 2.3.4.4, group A, Buan-like)^8^ was detected in wild birds and poultry in various countries of Asia, Europe, and—for the first time—North America. The global spread of HPAI H5N8 virus in 2014/2015 raises the question whether this H5 virus was better adapted to wild birds (i.e., increased virus replication and transmission combined with decreased virulence, resulting in better virus survival in wild bird populations) than the HPAI H5 virus from before 2014/2015.

The long-distance spread of HPAI H5 GsGd virus from Asia to other continents is thought to occur via migratory birds or poultry trade. With respect to the role of wild birds, timing and geographical location of HPAI H5 GsGd virus detection in wild birds corresponded with timing and direction of wild bird migration from shared breeding grounds, where multiple major flyways diverge toward separate wintering grounds. For instance, the newly emerging HPAI H5N8 GsGd virus was detected along major flyways in Russia at the end of summer in 2014, followed in time by detection of wild birds and/or poultry in Europe and in North America at the end of fall in 2014. A large-scale genetic analysis of HPAI H5 GsGd viruses, combined with analyses of epidemiological and ornithological data, indicated that the main routes of long-distance geographical spread were most probably via the infected migratory birds^9^. Furthermore, experimental infection studies showed that ducks of different species infected with HPAI H5 GsGd virus may excrete virus without showing clinical signs. Despite HPAI H5 GsGd viruses being highly pathogenic to gallinaceous poultry, these viruses were not uniformly pathogenic to domestic ducks^10, 11^ or wild ducks of different species (including Eurasian wigeon [*Anas penelope*], common teal [*Anas crecca*], mallard [*Anas platyrhynchos*], and common pochard [*Aythya ferina*])^12, 13^. Also, HPAI H5 GsGd virus-specific antibodies have been detected in free-living wild ducks of several species in Asia and Europe suggesting that these birds survive HPAI H5 GsGd virus infection^8, 14, 15^. Nevertheless, most wild birds in which HPAI H5 GsGd viruses have been detected were moribund or dead, and so far, field observations did not clarify the variable effect of the HPAI H5 GsGd virus infection on individuals, host species, and populations. Thus, field observations as well as experimental studies provide evidence for migratory birds as long-distance vectors for some HPAI H5 GsGd viruses, but indicate high variation in pathogenicity of the HPAI H5 GsGd virus infection, depending on the virus clade, host species, and individual host.

In this study, we question whether the HPAI H5N8 virus clade 2.3.4.4 group A detected in Europe in 2014 has different virulence in different species of wild waterbirds than the HPAI H5N1 virus detected in Europe before 2014. We experimentally infected four species of wild ducks: Eurasian wigeon, common teal, mallard, and common pochard with an avian isolate of HPAI H5N8 virus from Europe obtained in 2014. We chose the ducks because they are abundant, migratory, and an important group in the epidemiology and the ecology of influenza A viruses in the wild. We chose these particular duck species because of their abundance, preference for freshwater habitats, geographical distribution, and migratory pattern spanning Asia, Europe, and Africa. Additionally, we chose these particular duck species as well as these methods of inoculation and analysis to enable a comparison with previous experimental infections of wild ducks with HPAI H5N1 virus^13, 16^.

## Results

A total of 32 ducks of four different species (eight birds per species) were inoculated experimentally: one species of diving duck belonging to the genus *Aythya* (common pochard) and three species of dabbling ducks belonging to the genus *Anas* (mallard, common teal, and eurasian wigeon). All four duck species became infected with HPAI H5N8 virus, 63% (20/32) of the ducks according to virus isolation and 100% (32/32) of the ducks according to RT-PCR (Table 1). The number of ducks that became infected according to virus isolation differed among species: Eurasian wigeons and common teals were more often found to be infected with HPAI H5N8 virus than mallards and common pochards. No clinical signs of the disease were observed in any of the ducks, including loss of body mass (Figure S1). In contrast, mortality was observed in all chickens infected with HPAI H5N8 virus (see “Experimental design”).

**Table 1:** Health status and virus excretion of 32 wild ducks and four domestic chickens experimentally infected with highly pathogenic avian influenza virus A/chicken/Netherlands/emc-3/2014 (H5N8) GsGd clade 2.3.4.4 (group A, Buan-like)

			No. of birds that excreted virus from^a^			
			Pharynx		Cloaca	
Common name (taxonomic name)	No. of birds	No. of birds with clinical signs	Virus isolation	PCR	Virus isolation	PCR
Eurasian wigeon (*Anas penelope*)	8	0	6	8	4	8
Common pochard (*Aythya ferina*)	8	0	5	8	0	8
Mallard (*Anas platyrhynchos)*	8	0	3	8	0	8
Common teal (*Anas crecca*)	8	0	6	8	3	8
Chicken (*Gallus gallus*)	4	4	4	4	4	4

The minimal detection limit of virus isolation was 0.5 TCID_50_/ml and minimal area under the curve (AUC) from day 0 to 4 post inoculation was 2; if the values were on or below the minimal detection limit, birds were listed here as negative in the virus isolation

^a^Between 0 and 4 days post inoculation (dpi)

**Fig. 1:**
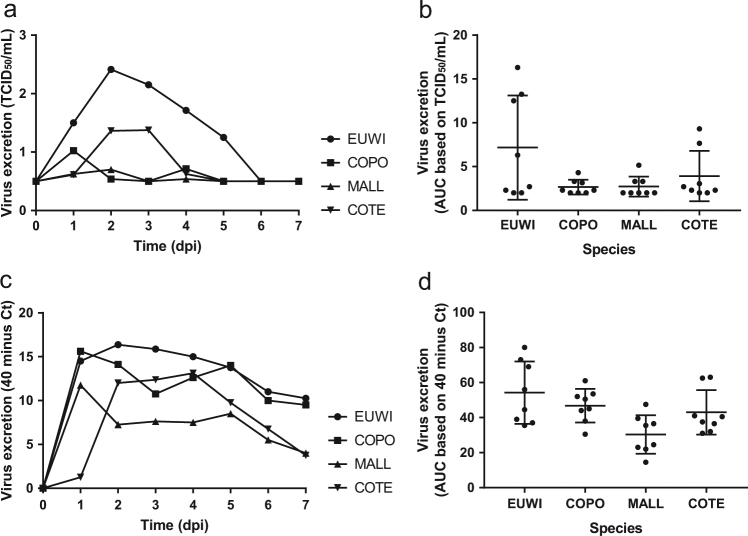
Mean virus excretion via the pharynx of the highly pathogenic avian influenza virus A/chicken/Netherlands/emc-3/2014 (H5N8) GsGd clade 2.3.4.4 (group A, Buan-like) of wild ducks, based on virus isolation (**a**, **b**) and virus detection by reverse transcription-PCR (RT-PCR) (**c**, **d**) of all birds in the group. Eurasian wigeon (EUWI, circle), common pochard (COPO, square), mallard (MALL, triangle pointed up), and common teal (COTE, triangle pointed down). TCID_50_, median tissue culture infectious dose with minimal detection limit of 0.5 TCID_50_/ml; Ct cycle threshold. AUC, area under the curve summarizes virus excretion from day 0 to 4 post inoculation (mean ± 95% confidence interval) based on virus isolation (**b**) and virus detection by RT-PCR (**d**)

Pharyngeal excretion of HPAI H5N8 virus varied significantly among the four duck species according to the virus isolation (one-way ANOVA of area under the curve (AUC) 0–4 dpi, *P* < 0.05) and RT-PCR (one-way ANOVA AUC 0–4 dpi, *P* < 0.05) (Tables 1 and 2, Figs. 1, S2, S3). The mean quantity of virus excreted per group from 0 to 4 dpi, according to the virus isolation and RT-PCR, was highest for Eurasian wigeon, followed by common teal, and was lowest for mallard and common pochard. According to virus isolation, pharyngeal excretion also varied substantially among individuals within the species (no statistical test performed) (Table S1, S2).

HPAI H5N8 virus that was excreted from the pharynx likely originated from the respiratory tract, based on virus isolation, immunohistochemistry (IHC), and RT-PCR. Infectious virus was isolated from the air sacs, trachea, and/or lung at 4 dpi. Specifically, infectious virus was isolated from the air sac (both common pochard and common teal), from trachea (common pochard), and lung (common teal) at 4 dpi (Table S1). Based on IHC, the air sacs and/or the nasal turbinates showed evidence of virus replication at 4 dpi. One common pochard (bird no. 11) demonstrated the presence of antigen in the epithelium of the air sac, and one common teal (bird no. 29) demonstrated presence of antigen in both the epithelium of the air sac and in mononuclear cells in the nasal turbinates. Based on histopathological examination of HE-stained slides, the presence of antigen in both birds was associated with slight infiltration of macrophages, plasma cells and lymphocytes, and a few heterophils, while in the common pochard, the presence of antigen was also associated with severe epithelial necrosis. By RT-PCR, the air sac most frequently had the lowest Ct value among all tissues per bird: ten times, compared to five times for trachea (Table S2). These two highest scoring tissues are both part of the respiratory tract. Remarkably, despite the frequent isolation of HPAI H5N8 virus from the pharyngeal samples collected from Eurasian wigeons, none of the respiratory tissues of these animals had histopathologic or immunohistochemical evidence of HPAI H5N8 virus replication at 4 dpi (Table S3).

**Fig. 2:**
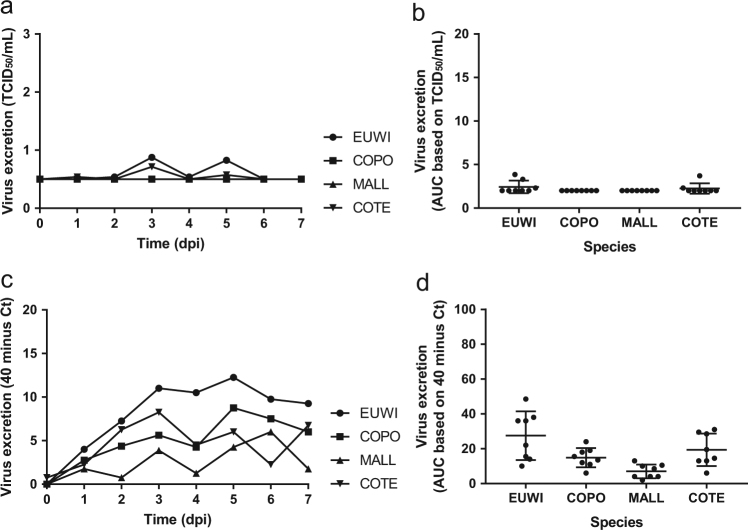
Mean virus excretion via cloaca of the highly pathogenic avian influenza virus A/chicken/Netherlands/emc-3/2014 (H5N8) GsGd clade 2.3.4.4 (group A, Buan-like) of wild ducks, based on virus isolation (**a**, **b**) and virus detection by reverse transcription-PCR (RT-PCR) (**c**, **d**). Legend same as for Fig. 1

For each of the four duck species, pharyngeal excretion of HPAI H5N8 virus exceeded cloacal excretion according to RT-PCR (paired *t*-test, *P* < 0.0003). According to virus isolation, pharyngeal excretion exceeded cloacal excretion for Eurasian wigeon only (paired *t*-test, *P* < 0.05). However, virus titers were low for the other duck species (Tables 1 and 2, Figs. 1 and 2).

Cloacal excretion of HPAI H5N8 virus was rare according to virus isolation (7/32, 22%) and limited to Eurasian wigeons (4/8) and common teals (3/8) (Table 1). However, cloacal virus excretion was frequent according to RT-PCR (32/32, 100%). The mean quantity of virus excreted from the cloaca per group did not differ among species according to virus isolation (Kruskal–Wallis test, AUC 0–4 dpi, *P* > 0.05), but did differ according to RT-PCR (one-way ANOVA AUC 0–4 dpi, *P* < 0.01) (Table 2, Figs. 2, S2, S3). Based on RT-PCR data of the mean quantity of virus excreted from the cloaca, Eurasian wigeons excreted significantly more virus from the cloaca compared to both mallard (*t*-test, *P* < 0.05) and common pochard (*t*-test, *P* < 0.05), but not compared to the common teal (*t*-test, *P* > 0.05). The common teals excreted significantly more virus from the cloaca compared to mallard (*t*-test, *P* < 0.05), but not compared to the common pochard (*t*-test, *P* > 0.05). According to virus isolation, and similar to pharyngeal excretion, cloacal excretion varied substantially among individuals within the species (Table S1, S2).

**Table 2:** Level and duration of virus excretion of the highly pathogenic avian influenza virus A/chicken/Netherlands/emc-3/2014 (H5N8) GsGd clade 2.3.4.4 (group A, Buan-like) from the pharynx and cloaca in four duck species, based on virus isolation

	Height and duration of virus excretion per route							
	Pharynx				Cloaca			
Common name (taxonomic name)	AUC (mean ± SEM)	Median (dpi)	Peak (dpi)	Range (dpi)	AUC (mean ± SEM)	Median (dpi)	Peak (dpi)	Range (dpi)
Eurasian wigeon (*Anas penelope*)	7.2 ± 2.1	2.5	2.5	0–8	2.4 ± 0.3	1	3.5	0–5
Common pochard (*Aythya ferina*)	2.7 ± 0.3	1	1	0–4	2 ± 0	0	0	0
Mallard (*Anas platyrhynchos)*	2.7 ± 0.4	0	2	0–4	2 ± 0	0	0	0
Common teal (*Anas crecca*)	3.9 ± 1.0	2	2.5	0–4	2.3 ± 0.2	0	3	0–5

The minimal detection limit of virus isolation was 0.5 TCID_50_/ml and minimal area under the curve (AUC) from day 0 to 4 post inoculation was 2. AUC, area under the curve summarizes infectious virus excretion from day 0 to 4 post inoculation; dpi, days post inoculation

HPAI H5N8 virus excreted from the cloaca may have originated from the kidney, pancreas, and/or liver, as infectious virus was isolated from these organs at 4 dpi (Table S1, birds no. 11 and 29). Virus produced in the kidney, pancreas, or liver could potentially have reached the cloaca via the urinary, pancreatic, or bile ducts. At 4 dpi, no immunohistochemical evidence for HPAI H5N8 virus replication was found in the kidney, pancreas, liver, or digestive tract. At this time point, there was limited histopathologic evidence for HPAI H5N8 virus replication, consisting of infiltration of mononuclear cells and few heterophils in the liver, and multifocally mild increase in monocytes or multifocal mild degeneration of tubular epithelial cells in the kidney. At 10 dpi, influenza virus antigen was detected by IHC in the pancreas of Eurasian wigeon no. 8. This was associated with multifocally coalescing and severe necrosis of the pancreatic parenchymal cells, and infiltration of many lymphocytes, moderate number of plasma cells, macrophages, and few heterophils. Overall, evidence of HPAI H5N8 virus replication in ducks at 4 dpi (and 10 dpi) was limited to tissues of the respiratory tract and tissues in direct connection with the digestive tract (i.e., kidney, pancreas, and liver).

In contrast to diagnosis of HPAI-H5N8-virus-associated encephalitis in chickens (see below), there was no evidence of HPAI H5N8 virus infection in the brain of any ducks according to IHC, histopathology, or virus isolation. Nonetheless, according to RT-PCR, weak positive values (Ct values ranged from 30 to 35) were found in the brains of Eurasian wigeon (1 of 4), common pochard (1 of 4), and common teal (3 of 4) (Table S1–S3).

Both naturally infected and experimentally infected chickens with HPAI H5N8 virus suffered from a systemic infection. In chickens in our study, HPAI H5N8 virus excretion took place both from the cloaca and from the oropharynx (Table 1, Table S2), similar as described before for other HPAI H5 GsGd virus clades^13, 17^. In contrast to the experimentally infected ducks, HPAI H5N8 virus was isolated from most tissues of experimentally or naturally infected chickens (Table S1). These tissues were of the brain, trachea (only experimentally, naturally not investigated), lung, air sac, heart, liver, spleen, kidney, small intestine, pancreas, and large intestine.

In experimentally infected chickens, evidence of virus replication by IHC was found in all sampled tissues except for the pectoral muscle (Table S3). Antigen was predominantly found in endothelial cells and in mononuclear inflammatory cells in these tissues. Additional cell types that demonstrated the presence of antigen were as follows: bronchial epithelial cells, air sac epithelial cells, cardiomyocytes, hepatocytes, glomerular and tubular epithelial cells in the kidney, esophageal epithelial cells, pancreatic acinar cells, neuronal and meningeal cells in the brain, glandular and lining epithelial cells in the infundibulum of the oviduct, and mesothelial cells in the serosa of various organs. The presence of antigen was mostly associated with histologic lesions consisting of necrosis, interstitial edema and hemorrhage, and inflammatory infiltrates with variable numbers of macrophages, plasma cells, lymphocytes, and heterophils.

In naturally infected chickens, evidence of virus replication by IHC was found in all tissues examined (Table S3). Remarkably, no evidence of virus replication by IHC was found in the small or large intestinal epithelial cells. Both the cell types positive for antigen by IHC as well as the associated lesions were comparable to those seen in the experimentally infected chickens, with most antigen found in endothelial cells and histologic lesions consisting of necrosis and mixed inflammatory infiltrates with variable numbers of macrophages, plasma cells, lymphocytes, and heterophils.

## Discussion

The results of this study on HPAI H5N8 virus infections in four duck species provided answers to the questions posed above. All three dabbling duck species (Eurasian wigeon, common teal, mallard) and the diving duck species (common pochard) excreted HPAI H5N8 virus in the absence of debilitating disease and therefore may act as long-distance vectors for HPAI H5N8 virus (clade 2.3.4.4 group A, Buan-like) as detected in Europe in 2014. The absence of disease in common pochards from this HPAI H5N8 virus in 2014 is in contrast to previous findings based on experimental inoculation of common pochards with HPAI H5N1 GsGd virus (clade 2.2.1)^13^.

Of the four duck species investigated here, the Eurasian wigeon is the most likely candidate as long-distance vector of HPAI H5N8 GsGd virus as infection was not associated with disease and virus excretion was most abundant in this species. Further support for Eurasian wigeons as a long-distance vector of HPAI H5N8 virus comes from the detection of H5N8-specific antibodies in blood samples collected in Korea and the Netherlands^8, 15^, indicating both virus circulation in the population and the ability to survive infection. In addition, HPAI H5N8 GsGd viruses (clade 2.3.4.4, group A) were isolated from apparently healthy free-living Eurasian wigeons sampled in Russia in 2014^18^, and in the Netherlands in 2014 and in 2015^15, 19^. Eurasian wigeons are strongly migratory, and make use of multiple major migratory flyways^20^. There are at least three wintering populations of Eurasian wigeons: Western Europe/Mediterranean/Africa, Northern India, and South-East Asia. The majority of these populations breed in northern Russia, and it is not clear to which degree their breeding areas overlap^19, 21, 22^.

In common teals, HPAI H5N8 virus excretion was lower than that in Eurasian wigeons. The virus replication, as found here in common teals, is in line with the findings in live birds sampled in Europe and Korea in 2014. HPAI H5N8 virus was detected in a swab from an apparently healthy common teal sampled in Germany in 2014 (sample type not specified)^23, 24^. Serological findings in free-living common teals sampled in Korea in 2014 provided evidence for survival of the infection with HPAI H5N8 virus (clade 2.3.4.4, group A), ancestral to HPAI H5N8 virus as detected in Europe in 2014^8^. Common teals are strongly migratory and their wintering populations cover a large range: southern Europe, southern Asia, and Africa^25^. Despite lower virus excretion, this species cannot be excluded as a long-distance vector for HPAI H5N8 virus.

HPAI H5N8 virus excretion was relatively low in mallards, which was suggested as a prime candidate for long-distance vector of HPAI H5N1 virus^12, 13^. Nevertheless, HPAI H5N8 GsGd viruses had been detected in hunter-harvested apparently healthy mallards sampled in Germany in December 2014 and January 2015^26^. Ancestral to HPAI H5N8 virus as detected in Europe in 2014^8^. Mallard wintering populations range from Europe to Southeast Asia^25^. Hence, mallard as a species may act as long-distance vector—either directly, or indirectly via linked shorter-distance migratory populations^27^—for HPAI H5N8 viruses.

In addition to the dabbling ducks, the diving duck species investigated here—common pochard—became infected without evidence of the disease. In 2014/2015, HPAI H5N8 virus was not detected in common pochards sampled alive or found dead in Europe or Asia. However, in total, just one live bird and no dead birds were sampled in the field^15^ and could therefore not be compared with our findings. Common pochards are migratory and winter in southern Europe, Africa, and southern Asia^25^. The absence of disease from this HPAI H5N8 virus in 2014 is in contrast with the previous findings from HPAI H5N1 virus, based on experimental inoculation of common pochards with HPAI H5N1 GsGd virus (clade 2.2.1), which resulted in mild to severe neurological disease^13^, and the detection of HPAI H5N1 GsGd virus in free-living common pochards found dead in Europe (i.e., France, Germany, and Switzerland) in 2006 (clade 2.2/2.2.1)). At that time, the virus also was detected in one apparently healthy common pochard^28^.

The pattern of HPAI H5N8 virus infection in the duck species investigated here was similar to that shown previously for HPAI H5 GsGd virus infection in dabbling ducks and diving ducks. Similar to earlier findings in dabbling and diving ducks, HPAI H5 virus excretion from the pharynx was higher than that from the cloaca^4, 12, 13, 17, 29, 30^. Based on a combination of RT-PCR, virus isolation, and IHC, the respiratory tract is the most likely source for the virus excreted through the pharynx. In chickens, a combination of RT-PCR, virus isolation, and IHC implied systemic virus replication (Table S1-3). Despite some HPAI H5 virus excretion from the cloaca, no evidence was found in this study for HPAI H5 virus replication in the intestinal epithelium, which is similar to the lack of evidence for HPAI H5 virus replication in the intestinal epithelium in the following Eurasian migratory duck species: common pochard, common teal, Eurasian wigeon, gadwall (*Anas strepera*) (at 4 dpi^13^), mallard (at 4 dpi^13, 30^; at 3 dpi^17^), and tufted duck (*Aythya fuligula*) (at 3 or 4 dpi^13, 16^). In our study, the lack of evidence for HPAI virus replication in the intestinal epithelium at 4 dpi corresponds with the absence of infectious virus excretion from the cloaca at 4 dpi in 16 birds from which the tissues were taken. Most ducks excreted infectious HPAI H5 virus from the cloaca at 3 dpi (1 dpi 1/32 ducks, 2 dpi 1/32, 3 dpi 3/32, 4 dpi 1/32); therefore the time point for IHC at 4 dpi may have been too late to identify the location of virus replication. However, based on virus isolation from tissues and evidence of virus replication by IHC at 4 dpi, the kidney, pancreas, and/or liver are the most likely source for virus excreted through the cloaca. This is in contrast to most LPAI viruses, for which the epithelium lining of the intestinal tract is the main replication site^31–33^. Thus, also for this HPAI H5N8 virus, the main route of excretion in ducks seems to be from the pharynx.

Experimental infections with HPAI H5 GsGd viruses show differences in virulence within and between different duck species. In dabbling ducks, no evidence of disease was shown as a result of HPAI H5N8 GsGd virus (clade 2.3.4.4 group A) infection, as has been shown previously for HPAI H5N1 GsGd virus infections in subadult dabbling ducks of the species gadwall, Eurasian wigeon, common teal^13^, northern pintail (*Anas acuta*)^12^, and mallard^12, 13, 29^ (Table S4). In diving ducks, no evidence of disease was shown as a result of HPAI H5N8 GsGd virus infection (clade 2.3.4.4 group A) in common pochard, in contrast to the historical findings upon experimental inoculation of the same species with HPAI H5N1 GsGd virus^13^. This suggests that HPAI H5N8 GsGd virus (clade 2.3.4.4 group A) has a lower virulence for common pochards than HPAI H5N1 GsGd virus. Nevertheless, in 2016/2017 HPAI H5N8 GsGd clade 2.3.4.4 viruses of a different genetic group (i.e., group B, Gochang-like)^8^ emerged in wild birds and poultry in Europe associated with mass die-offs among wild birds (including Eurasian wigeons)^34^. Experimental infection of wild birds, according to a similar protocol as above, would allow evaluation of possible changes in the susceptibility to infection and virulence of HPAI H5 GsGd clade 2.3.4.4 group B compared to group A.

The results of this study provide the strongest evidence for Eurasian wigeons as long-distance vectors for HPAI H5N8 virus (clade 2.3.4.4 group A), although common teals, mallards, and common pochards cannot be excluded. This switch in level of virus excretion—highest in Eurasian wigeons for the 2014 virus, compared to highest for common pochards and mallards for the 2005 virus—and the decreased severity of clinical disease—no clinical disease in any of the birds infected with the 2014 virus, compared to over 50% clinical disease and pathological changes in common pochards infected with the 2005 virus—implies that the HPAI H5 GsGd viruses have adapted to wild waterbird populations over time. Whether this means that the observed changes allow more efficient long-distance virus transport in Eurasian wigeons is yet to be determined. Also, only a very limited number of waterbird species were examined in this study, and it cannot be excluded that other waterbird species are involved in HPAI H5 epidemiology. Further studies that combine laboratory evaluation of HPAI viral virulence and transmissibility in wild waterbirds and field studies on the spread of HPAI virus in wild waterbirds, as well as on the spatial and temporal connectivity between their migratory flyways, are essential to clarify the changing role of wild waterbirds in the epidemiology of HPAI H5 GsGd virus infections.

## Materials and methods

### Virus preparation

The HPAI H5N8 virus of the clade 2.3.4.4 used in this study was isolated from the lung homogenate of a naturally infected chicken during an outbreak at a farm in Ter Aar, the Netherlands, in 2014 (A/chicken/Netherlands/emc-3/2014). Full-genome sequence of this virus is available in the GenBank under the following accession number: KR233687 to KR233694. The virus was propagated by two passages in Madin–Darby canine kidney (MDCK) cells. The harvested supernatant had a titer of 1 × 10^7.3^ median tissue culture infectious dose (TCID_50_)/ml and was diluted with phosphate-buffered saline (PBS) to 1 × 10^4^ TCID_50_/3 ml. This virus was chosen due to its high pathogenicity in naturally infected chickens and for consistency to compare the findings with ferrets experimentally infected intranasally with the same H5N8 virus^35^. All experiments with HPAI H5N8 virus were performed under Animal Biosafety Level 3 conditions.

### Animals

Ducks of four different species were included in this study: common pochard (diving duck, belonging to the genus *Aythya*), mallard, common teal, and Eurasian wigeon (dabbling ducks, belonging to the genus *Anas*). For each species, males and females were equally represented. All ducks used for the infection experiment were captive-bred (breeding farm Man in ‘t Veld, Klarenbeek, the Netherlands). Both chicken and ducks arrived in the lab 11 days prior to the day of inoculation, were in good condition, and looked apparently healthy. Birds were moved into isolators 3 days prior to the day of inoculation. Ducks were approximately 1 year of age at the time of inoculation. Blood samples (−3 days post inoculation [dpi]), cloacal swabs, and pharyngeal swabs (−3 dpi and 0 dpi) were collected from all ducks before inoculation. Serum was analyzed for the detection of antibodies specific to influenza A virus (nucleoprotein, NP), using a commercial blocking enzyme-linked immunosorbent assay (bELISA) (IDEXX Laboratories B.V., Hoofddorp, the Netherlands) and according to the manufacturer’s instructions. Swabs were analyzed for the detection of influenza A viruses by a reverse transcription-polymerase chain reaction (RT-PCR) targeting the matrix gene. No NP-specific serum antibodies were detected. All ducks were tested negative for virus detection by RT-PCR for the matrix gene, except for three ducks (i.e., one mallard at −3 dpi and two common teals at 0 dpi). These birds tested weakly positive for virus by RT-PCR for the matrix gene (Ct values ranged from 32 to 38) and one of the two common teals tested weakly positive for H5 virus detection by RT-PCR (Ct value 35). No influenza A viruses were isolated from these samples upon inoculation into the 11-day-old embryonated chicken eggs, all three ducks subsequently became infected with HPAI H5N8 virus after inoculation, and two of these three ducks showed the highest virus excretion of their species. Birds had access to food and water ad libitum. Prior to the experiment, ducks were housed together in one animal room. A total of four specific-pathogen-free White Leghorn chickens of 14 months of age at the time of inoculation were used as positive controls for the pathogenicity of the virus stock. Chickens were obtained from the Animal Health Service, Deventer, the Netherlands. Neither NP-specific antibodies by bELISA, nor influenza A viruses by RT-PCR for the matrix gene were detected in chickens prior to the experiment at −3 and 0 dpi.

### Experimental design

For each duck species, eight birds were housed together in negatively pressured class 3 isolators. Chickens were housed with four birds together in a negatively pressured class 3 isolator. A volume of 3 mL containing 1 × 10^4^ TCID_50_ HPAI H5N8 virus was inoculated into each bird, 1.5 ml into the trachea, and 1.5 ml into the esophagus. The cloacal and pharyngeal samples were collected daily from 0 to 14 dpi and on 16 and 18 dpi using sterile cotton swabs and each placed in 1 ml virus transport medium^36^. Samples for virus detection were stored at −80 °C from within 2 h of collection until analysis.

Each group of eight birds was randomly divided into two groups of four birds. One group was euthanized by exsanguination under isoflurane anesthesia for pathologic examination at 4 dpi; the other group was monitored for virus excretion until 18 dpi. Due to the development of ulcers on the wings of two common teals (bird nos. 29 and 31) at 2–3 dpi—not related to the HPAI H5N8 virus infection, but caused by trauma—we decided to euthanize these two birds at 4 dpi instead of at 18 dpi, as initially determined by randomization. Therefore, the common teals were no longer randomly divided into two groups of birds. In addition, two birds—both supposed to be euthanized at 18 dpi—had to be removed from the experiment at 10 dpi due to lethargy (Eurasian wigeon no. 8) or at 11 dpi due to luxation of the left leg (common pochard no. 16). No viruses were isolated from the tissues and cloacal or pharyngeal swabs of these birds at the day of removal from the experiment.

As expected, by 2 dpi 100% of the positive-control chickens were dead. In addition to comparing the findings of experimentally infected ducks with those of experimentally infected chickens, we also compared them with the findings of five H5N8-positive chickens found dead that were collected during the same outbreak as from which the virus strain A/chicken/Netherlands/emc-3/2014 (H5N8) was isolated. The study was approved by an independent animal experimentation ethical review committee, approved by the Dutch government (Stichting DEC consult) (permit number 122-15-01 and 122-15-02).

### Virus detection and titration

Tissues collected for virus detection and titration were the brain, trachea, lung, air sac, pancreas, liver, jejunum, colon, heart, spleen, and kidney. Tissues were weighed and homogenized in 1 ml virus transport medium using a homogenizer (Fastprep®-24, MP Biomedicals, Santa Ana, California, USA). RNA isolation and RT-PCR of the tissue samples and cloacal and pharyngeal swabs were performed as described previously^37^. Briefly, RNA from the tissue and swab suspensions was isolated using a MagNaPure LC System with the MagNaPure LC Total Nucleic Acid Isolation Kit (Roche Diagnostics, Almere, the Netherlands). RT-PCR were performed on an ABI 7700 machine (Applied Biosystems, Foster City, CA, USA) using a TaqMan Fast Virus 1-Step Master Mix (Applied Biosystems). The oligonucleotides (5′-CTT-CTR-ACC-GAG-GTC-GAA-ACG-TA-3′) and (5′ -TCT-TGT-CTT-TAG-CCA-YTC-CAT-GAG-3′) and the labeled probes (5′ 6-FAM-TCA-GGC-CCC-CTC-AAA-GCC-GAG-A-BHQ-3′) and (5′ 6-FAM-TCA-GGC-CCC-CTC-AAA-GCC-GAA-A-BHQ-3′) were used for detection of the matrix segment of influenza A viruses. Samples were considered virus positive if the cycle threshold (Ct) value was <40.

Virus titers were shown as per gram tissue and determined in triplicate based on 10-fold serial dilutions of the homogenized tissue samples and swabs on MDCK cells, as described^38^. To compare the virus culture in MDCK cells and in embryonated chicken eggs, aliquots of a cloacal swab and a pharyngeal swab collected from each of the eight Eurasian wigeons at 3 dpi were inoculated in parallel into the MDCK cells and into the 11-day-old embryonated chicken eggs. All 16 samples were tested positive in the matrix-RT-PCR (Ct values ranged from 17 to 38). From three of the eight Eurasian wigeons, no virus was cultured using either method; from the remaining five Eurasian wigeons, virus was cultured from six of the ten samples using both methods, and the virus was cultured from another two samples exclusively using embryonated chicken eggs.

### Histopathology and immunohistochemistry

At 4 dpi, the samples of the following tissues were collected from ducks for histopathology and IHC: nasal turbinates, brain, trachea, lung, air sac, pancreas, liver, esophagus, proventriculus, duodenum, jejunum, ileum, colon, cecum, heart, spleen, kidney, adrenal glands, gonads, bursa, and pectoral muscle. At 1 or 2 dpi, the same tissues plus oviduct and wattle were collected from chickens. For the naturally infected chickens, the same tissues were collected, except for the pectoral muscle, gonads, adrenal, duodenum, and ileum. The tissue samples were fixed in 10% neutral-buffered formalin, embedded in paraffin, and cut into 4-μm-thick sections. For histopathologic examination, the tissue sections were stained with hematoxylin and eosin (HE). For detection of cells expressing influenza A virus antigen, the tissue sections were subjected to IHC analysis with a monoclonal antibody against the nucleoprotein of influenza A virus as primary antibody^39^.

### Clinical signs of infection

Body mass was monitored daily from 0 to 10 dpi, and on 12, 14, 16, and 18 dpi. Each morning before handling the birds, the individual birds in each group were scored quantitatively for nervous and other clinical signs. Nervous signs included walking toward a person, unsure tread, circling, falling over, tremors, and torticollis. Other clinical signs included ruffled feathers, decreased movement or feeding activity, exudate from eyes or nose, labored breathing, and lethargy.

### Statistical analyses

To compare virus excretion among and within groups, the AUC of infectious virus (based on virus isolation) or viral RNA (based on RT-PCR) from 0 to 4 dpi was calculated. The mean quantity of virus excreted per group from the cloaca or pharynx (i.e., mean AUC) was based on the AUC of all birds in the group. To compare the duration of virus excretion among and within groups, the median of maximum day of infectious virus presence (i.e., positive virus isolation) was used. The median duration of virus excretion per group was based on the values of all birds in the group. To investigate whether the differences in virus excretion or duration were statistically significant between routes of virus excretion (i.e., cloaca and pharynx), a paired *t*-test or Wilcoxon test was performed. To investigate whether the differences in virus excretion or duration were statistically significant among species, a one-way ANOVA or Kruskal–Wallis test was performed.

## Electronic supplementary material

Figure S1 Body mass of individual ducks in time (0–10, 12, 14 dpi). Eurasian wigeon (A); common pochard (B); mallard (C); common teal (D)

Figure S2 Virus excretion of individual birds based on virus isolation of highly pathogenic avian influenza virus A/chicken/Netherlands/emc-3/2014 (H5N8) GsGd clade 2.3.4.4 (group A, Buan-like) via the pharynx (A, C, E, G) and via cloaca (B, D, F, H), with minimal detection limit of 0.5 TCID_50_/ml

Figure S3 Virus excretion of individual birds based on RT-PCR detection of highly pathogenic avian influenza virus A/chicken/Netherlands/emc-3/2014 (H5N8) GsGd clade 2.3.4.4 (group A, Buan-like) via the pharynx (A, C, E, G) and via cloaca (B, D, F, H)

Table S1

Table S2

Table S3

Table S4
