# Supplementary figures and images for: Wild ducks excrete highly pathogenic avian influenza virus H5N8 (2014–2015) without clinical or pathological evidence of disease
Source: Emerg Microbes Infect. 2018 Apr 18;7:67. doi: 10.1038/s41426-018-0070-9 (PMC5906613; doi:10.1038/s41426-018-0070-9)

# Figure S1

a

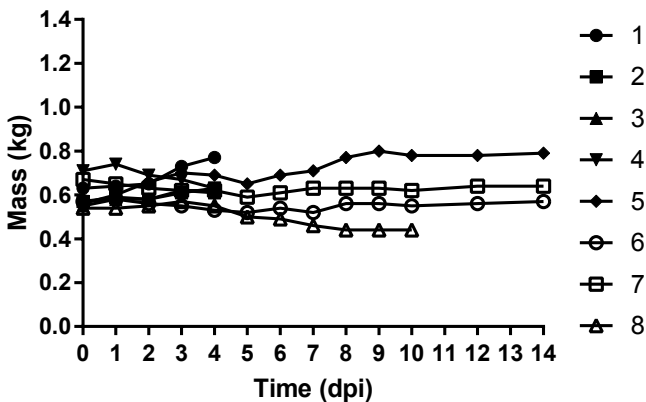

**b**

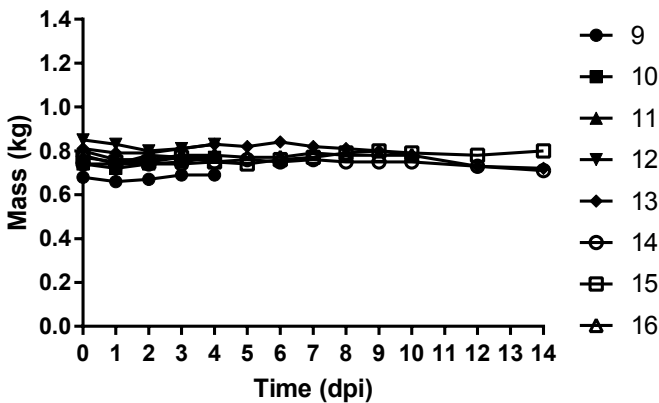

C

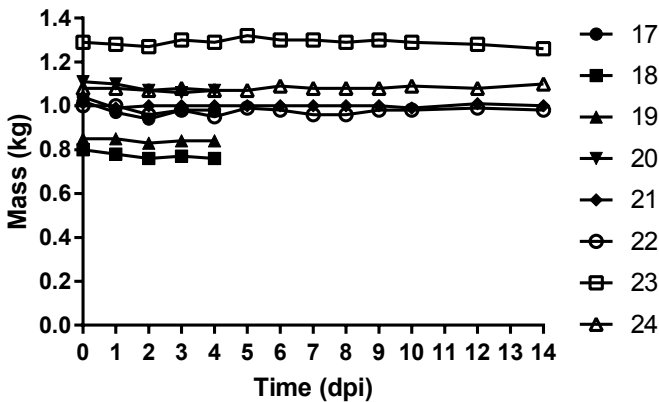

**d**

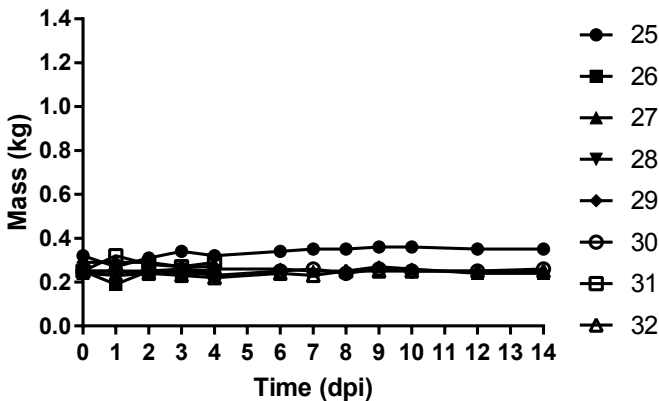

Supplement: Supplementary file 1 — Figure S1 Body mass of individual ducks in time (0–10, 12, 14 dpi). Eurasian wigeon (A); common pochard (B); mallard (C); common teal (D) [file 41426_2018_70_MOESM1_ESM.pdf]

## Figure S2

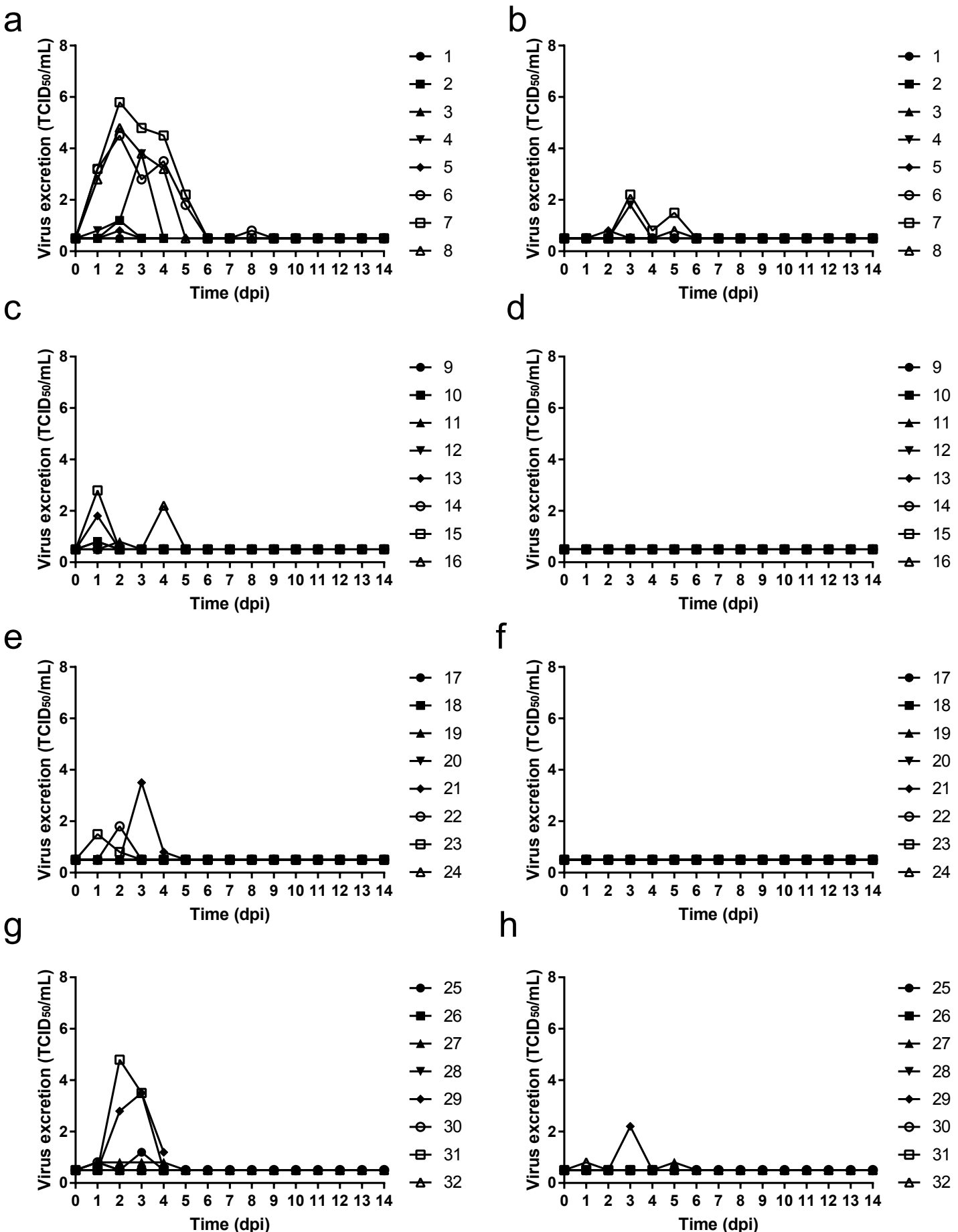

Supplement: Supplementary file 2 — Figure S2 Virus excretion of individual birds based on virus isolation of highly pathogenic avian influenza virus A/chicken/Netherlands/emc-3/2014 (H5N8) GsGd clade 2.3.4.4 (group A, Buan-like) via the pharynx (A, C, E, G) and via cloaca (B, D, F, H), with minimal detection limit of 0.5 TCID50/ml [file 41426_2018_70_MOESM2_ESM.pdf]

Figure S3

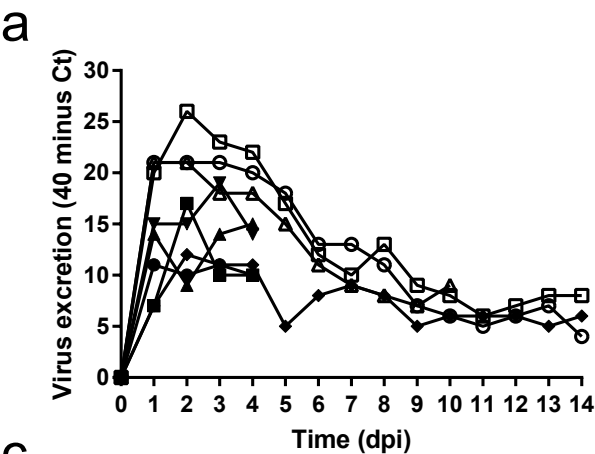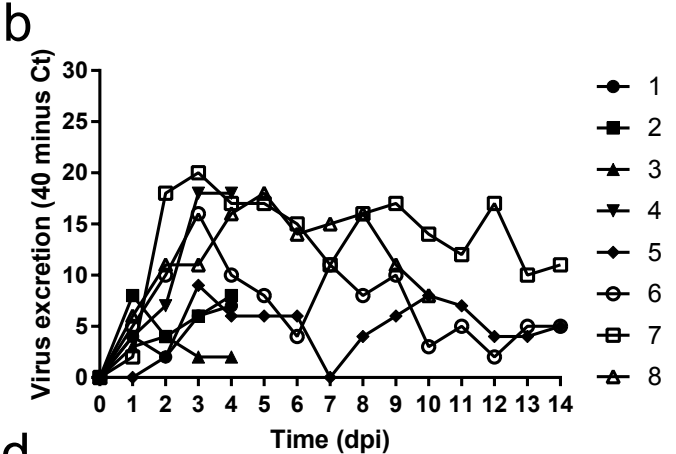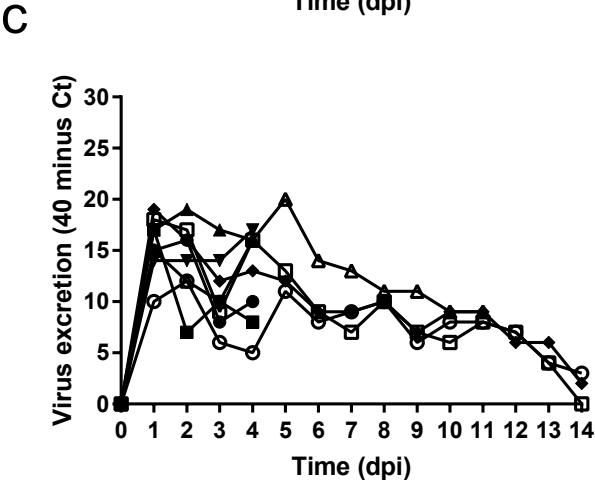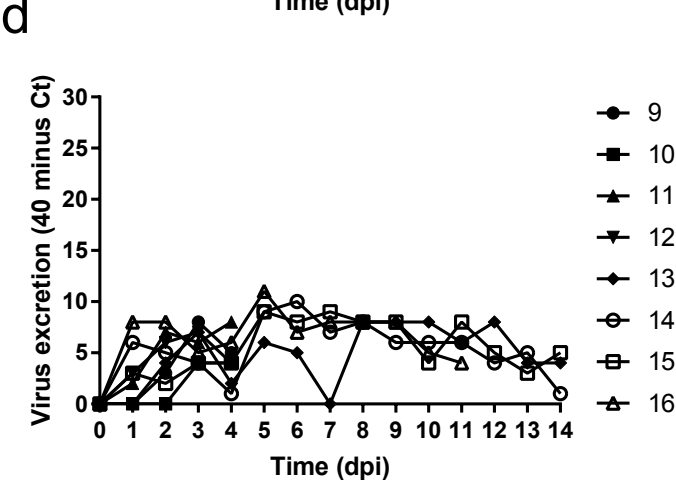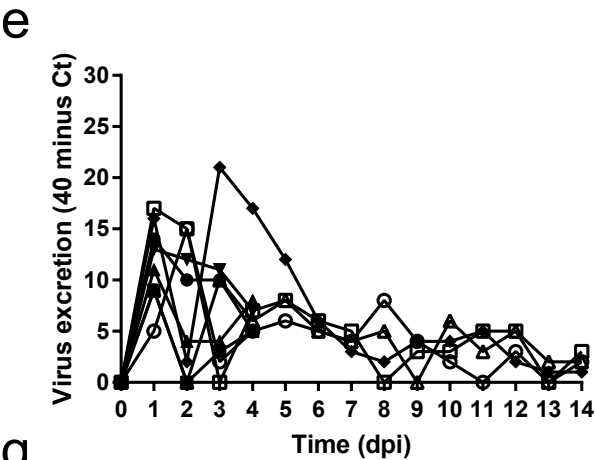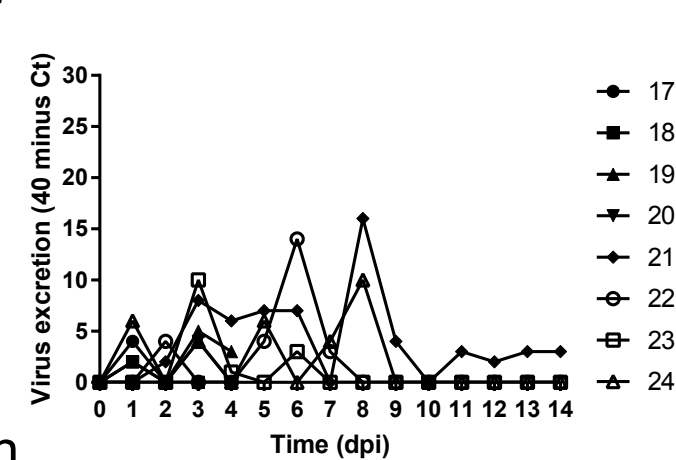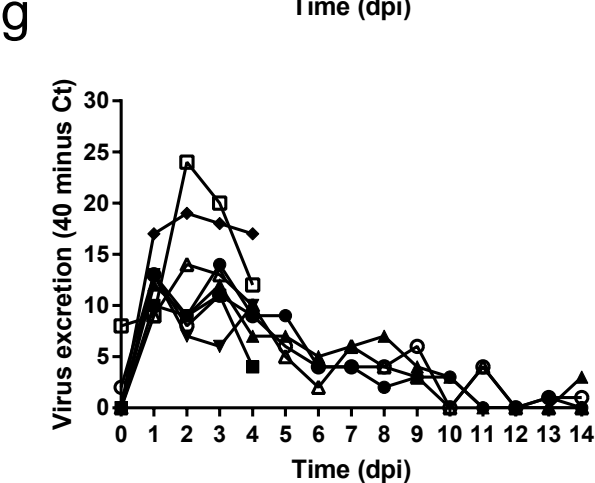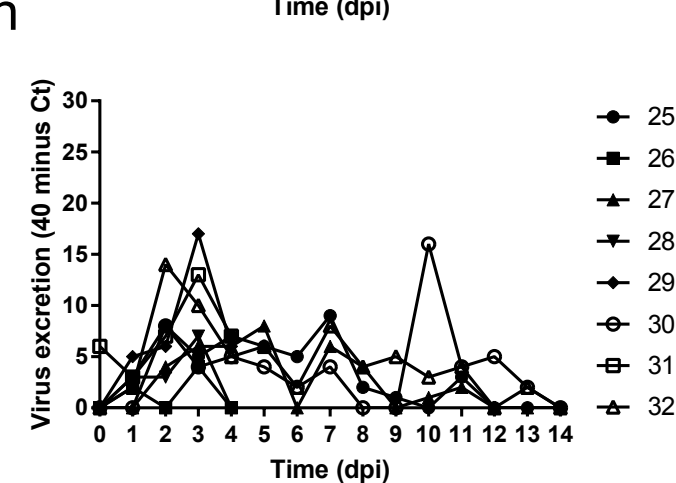

Supplement: Supplementary file 3 — Figure S3 Virus excretion of individual birds based on RT-PCR detection of highly pathogenic avian influenza virus A/chicken/Netherlands/emc-3/2014 (H5N8) GsGd clade 2.3.4.4 (group A, Buan-like) via the pharynx (A, C, E, G) and via cloaca (B, D, F, H) [file 41426_2018_70_MOESM3_ESM.pdf]
