# Supplementary material for: Wild ducks excrete highly pathogenic avian influenza virus H5N8 (2014–2015) without clinical or pathological evidence of disease
Source: Emerg Microbes Infect. 2018 Apr 18;7:67. doi: 10.1038/s41426-018-0070-9 (PMC5906613; doi:10.1038/s41426-018-0070-9)
Supplement: Supplementary file 4 — Table S1 [file 41426_2018_70_MOESM4_ESM.pdf]

Table S1. Isolation of highly pathogenic avian influenza virus A/chicken/Netherlands/eur-3/2014 (H5N8) from organs of wild ducks at 4 days post inoculation (dpi), from organs of domestic chickens at 1 and 2 dpi (exp.) and upon natural infection (nat.), and from swabs at 1–4 dpi

		Virus titer (log TCID50 per g tissue)*																								
		Wild birds																Domestic birds								
		Eurasian wigeon no.				Common pochard no.				Mallard no.				Common teal no.				Chicken (exp.) no.**				Chicken (nat.) no.**				
System	Organ	1	2	3	4	9	10	11	12	17	18	19	20	26	28	29	31	33	34	35	36	GG.1	GG.2	GG.3	GG.4	GG.5
Nervous	Brain	-	-	-	-	-	-	-	-	-	-	-	-	-	-	-	-	3.5	3.4	5.1	5.6	2.3	-	3.6	5.1	4.2
Respiratory	Trachea	-	-	-	-	-	-	2.7	-	-	-	-	-	-	-	-	-	6.0	4.0	5.4	7.0	nd	nd	nd	nd	nd
	Lung	-	-	-	-	-	-	-	-	-	-	-	-	-	-	3.6	-	6.3	5.6	5.6	6.4	6.3	5.7	4.8	4.6	6.5
	Air sac	-	-	-	-	-	-	2.7	-	-	-	-	-	-	-	5.5	-	6.8	5.9	5.7	6.2	2.5	4.8	7.1	2.7	7.1
Digestive	Pancreas	-	-	2.0	-	-	-	-	-	-	-	-	-	-	-	-	-	-	2.2	3.2	-	4.9	4.2	3.2	-	-
	Liver	-	-	-	-	-	-	2.9	-	-	-	-	-	-	-	-	-	6.5	5.1	5.6	7.9	3.2	-	3.8	4.4	5.3
	Jejunum	-	-	-	-	-	-	-	-	-	-	-	-	-	-	-	-	5.5	7.2	7.2	6.7	3.7	5.6	5.5	5.2	6.2
	Colon	-	-	-	-	-	-	-	-	-	-	-	-	-	-	-	-	6.8	3.4	5.8	6.1	5.8	6,0	4.8	5.1	6.5
Other	Heart	-	-	-	-	-	-	-	-	-	-	-	-	-	-	-	-	5.4	4.1	4.5	6.6	2.7	1.9	2.3	4.2	5.3
	Spleen	-	-	-	-	-	-	-	-	-	-	-	-	-	-	-	-	7.2	6.8	5.4	7.5	4.6	5.1	4.8	5,0	6.5
	Kidney	-	-	-	-	-	-	1.7	-	-	-	-	-	-	-	1.5	-	6.1	5.9	5.4	7.0	5.3	5.6	-	3.8	7.5
Swab***	Cloaca, 1 dpi	-	-	-	-	-	-	-	-	-	-	-	-	-	-	-	-	-	-	-	-	nd	nd	nd	nd	nd
	Cloaca, 2 dpi	-	-	-	-	-	-	-	-	-	-	-	-	-	-	-	-	nd	nd	4.5	4.8	nd	nd	nd	nd	nd
	Cloaca, 3 dpi	-	-	-	1.8	-	-	-	-	-	-	-	-	-	-	2.2	-	nd	nd	nd	nd	nd	nd	nd	nd	nd
	Cloaca, 4 dpi	-	-	-	-	-	-	-	-	-	-	-	-	-	-	-	-	nd	nd	nd	nd	nd	nd	nd	nd	nd
	Cloaca																					2.5	5.2	3.5	1.8	5.2
	Pharynx, 1 dpi	-	-	-	0.8	-	0.8	-	-	-	-	-	-	-	-	-	-	2.5	-	-	2.2	nd	nd	nd	nd	nd
	Pharynx, 2 dpi	-	1.2	-	1.2	-	-	0.8	-	-	-	-	-	-	-	2.8	4.8	nd	nd	4.8	3.8	nd	nd	nd	nd	nd
	Pharynx, 3 dpi	-	-	-	3.8	-	-	-	-	-	-	-	-	-	-	3.5	3.5	nd	nd	nd	nd	nd	nd	nd	nd	nd
	Pharynx, 4 dpi	-	-	-	-	-	-	-	-	-	-	-	-	-	-	1.2	-	nd	nd	nd	nd	nd	nd	nd	nd	nd
	Pharynx																					4.8	2.5	5.2	4.8	5.8

*TCID50, median tissue culture infectious dose; white, no virus isolated; yellow, virus titer 0.8–1.9; orange, 2.0–3.9; red, 4.0–5.9; dark red, 6.0–7.9; dpi, days postinoculation.

**Chickens died at 1 day post inoculation (33 and 34) and 2 days postinoculation (35 and 36)

"-"=negative, equal to titer <0.5; nd = no data available

***Please note that results for virus excretion via the cloaca and pharynx as shown here are limited to the results of the 16 birds that were euthanized at 4 dpi and serve as a comparison with the findings of the tissues. Results of the remaining 16 birds are not shown here.
