# Supplementary material for: Wild ducks excrete highly pathogenic avian influenza virus H5N8 (2014–2015) without clinical or pathological evidence of disease
Source: Emerg Microbes Infect. 2018 Apr 18;7:67. doi: 10.1038/s41426-018-0070-9 (PMC5906613; doi:10.1038/s41426-018-0070-9)
Supplement: Supplementary file 5 — Table S2 [file 41426_2018_70_MOESM5_ESM.pdf]

Table S2. Detection of highly pathogenic avian influenza virus A/chicken/Netherlands/emc-3/2014 (H5N8) by reverse transcription–PCR from organs of wild ducks at 4 days postinoculation (dpi), from organs of experimentally infected domestic chickens at 1 and 2 dpi, and from swabs at 1–4 dpi.

		Viral RNA (cycle threshold value per g tissue)*																			
		Wild birds																Domestic birds			
		Eurasian wigeon no.				Common pochard no.				Mallard no.				Common teal no.				Chicken no.**			
System	Organ	1	2	3	4	9	10	11	12	17	18	19	20	26	28	29	31	33	34	35	36
Nervous	Brain	-	-	33	-	-	-	-	32	-	-	-	-	33	-	28	31	20	22	14	16
Respiratory	Trachea	-	24	25	21	20	29	23	25	27	25	27	24	21	24	21	27	14	15	11	11
	Lung	-	28	22	19	30	27	25	25	32	33	25	29	27	27	17	25	19	18	20	15
	Air sac	31	22	17	9	31	23	15	15	-	26	24	27	24	24	9	16	9	15	13	11
Digestive	Pancreas	-	-	28	28	31	33	29	32	-	-	34	35	-	27	25	33	17	15	16	15
	Liver	-	36	29	28	31	32	23	29	-	-	-	32	33	32	25	31	16	15	18	12
	Jejunum	-	-	25	22	-	32	30	29	-	-	30	31	31	28	21	30	14	17	15	11
	Colon	-	-	22	24	32	32	29	28	-	-	30	32	32	22	19	29	13	11	10	11
Other	Heart	-	-	32	30	33	31	29	32	-	-	-	-	30	34	23	31	14	16	9	10
	Spleen	-	-	30	21	25	26	28	26	-	30	30	30	26	22	19	22	13	14	15	11
	Kidney	37	-	22	25	32	30	25	30	33	-	33	25	32	27	25	32	18	17	13	13
Swab***	Cloaca, 1 dpi	36	32	37	36	-	-	38	37	36	38	38	-	38	37	35	37	31	39	31	36
	Cloaca, 2 dpi	38	36	36	33	37	-	33	34	-	-	-	-	-	37	34	33	nd	nd	17	19
	Cloaca, 3 dpi	34	34	38	22	32	36	34	33	-	-	35	36	36	33	23	27	nd	nd	nd	nd
	Cloaca, 4 dpi	33	32	38	22	35	36	32	36	-	-	37	-	-	-	34	33	nd	nd	nd	nd
	Pharynx, 1 dpi	29	33	26	25	25	23	23	26	26	31	29	27	30	27	23	31	21	26	28	22
	Pharynx, 2 dpi	30	23	31	25	24	33	21	26	30	-	36	28	31	33	21	16	nd	nd	18	22
	Pharynx, 3 dpi	29	30	26	21	32	30	23	26	30	37	36	29	29	34	22	20	nd	nd	nd	nd
	Pharynx, 4 dpi	30	30	25	26	30	32	24	23	35	35	32	33	36	30	23	28	nd	nd	nd	nd

*White, no virus detected; yellow, 30–39 Ct; orange, 20–29 Ct; red, 10–19 Ct; dark red, 1-9 Ct; dpi, days postinoculation.
**Domestic chickens died at 1 day postinoculation (33 and 34) and 2 days postinoculation (35 and 36)
***cycle treshold value per 200 microliter
"-"=negative, equal to titer <0.5; nd = no data available
