# Supplementary material for: Wild ducks excrete highly pathogenic avian influenza virus H5N8 (2014–2015) without clinical or pathological evidence of disease
Source: Emerg Microbes Infect. 2018 Apr 18;7:67. doi: 10.1038/s41426-018-0070-9 (PMC5906613; doi:10.1038/s41426-018-0070-9)
Supplement: Supplementary file 6 — Table S3 [file 41426_2018_70_MOESM6_ESM.pdf]

Table S3. Expression of antigen in organs of wild ducks at 4 days postinoculation (dpi) with highly pathogenic avian influenza virus A/chicken/Netherlands/eur-3/2014 (H5N8) and in organs of domestic chickens at 1 and 2 dpi (exp.) and upon natural infection (nat.)

		Antigen expression per species*																								
		Wild birds																Domestic birds								
		Eurasian wigeon no.**				Common pochard no.				Mallard no.				Common teal no.				Chicken (exp.) no.**				Chicken (nat.) no.**				
System	Organ	1	2	3	4	9	10	11	12	17	18	19	20	26	28	29	31	33	34	35	36	GG.1	GG.2	GG.3	GG.4	GG.5
Nervous	Brain	-	-	-	-	-	-	-	-	-	-	-	-	-	-	-	-	-	+/-	+	+	-	-	+	+/-	+/-
Respiratory	Nose	-	-	-	-	-	-	-	-	-	-	-	-	-	-	+/-	-	+	+	+	+	-	-	+	+	+
	Trachea	-	-	-	-	-	-	-	-	-	-	-	-	-	-	-	-	+/-	-	+	+	-	+/-	-	+	+
	Lung	-	-	-	-	-	-	-	-	-	-	-	-	-	-	-	-	+	+	++	++	+	+	+	+	+
	Air sac	-	-	-	-	-	-	+/-	-	-	-	-	-	-	-	+	-	++	++	+	++	+	+	+	+	+
Digestive	Esophagus	-	-	-	-	-	-	-	-	-	-	-	-	-	-	-	-	+	+	+	+	nd	nd	nd	nd	nd
	Proventriculus	-	-	-	-	-	-	-	-	-	-	nd	-	-	-	-	-	+	+/-	+	+	-	-	nd	-	+
	Duodenum	-	-	-	-	-	-	-	-	-	-	-	-	-	-	-	-	+	+	+	+	nd	nd	nd	nd	nd
	Pancreas**	-	-	-	-	-	-	-	-	-	-	-	-	-	-	-	-	+/-	+/-	+	+	+	+	+	+	+
	Liver	-	-	-	-	-	-	-	-	-	-	-	-	-	-	-	-	+/-	+/-	++	++	+/-	-	+/-	+/-	+/-
	Jejunum	-	-	-	-	-	-	-	-	-	-	-	-	-	-	-	-	+	+	+	+	+	+	+	+	+
	Ileum	-	-	-	-	-	-	-	-	-	-	-	-	-	-	-	-	+	+	+	+	nd	nd	nd	nd	nd
	Caecum	-	-	-	-	-	-	-	-	-	-	-	-	-	-	-	-	+	+	+	+	+	+	+	+	+
	Colon	-	-	-	-	-	-	-	-	-	-	-	-	-	-	-	-	+	+	+	+	+	+	+	+	+
Other	Bursa	-	-	-	-	-	-	-	-	-	-	-	-	-	-	-	-	nd	nd	nd	nd	nd	nd	nd	nd	nd
	Heart	-	-	-	-	-	-	-	-	-	-	-	-	-	-	-	-	+	+/-	++	++	+/-	-	+	+	+
	Pectoral muscle	-	-	-	-	-	-	-	nd	-	-	-	-	-	-	-	-	-	-	-	-	nd	nd	nd	nd	nd
	Spleen	-	-	-	-	-	-	-	-	-	-	-	-	-	-	-	-	+	+	+	+	+	-	+	+	+
	Kidney	-	-	-	-	-	-	-	-	nd	-	-	-	-	-	-	-	-	+/-	+	+	-	-	nd	-	+
	Adrenal	nd	nd	-	-	-	-	-	-	-	-	nd	-	-	-	-	-	+	nd	nd	nd	nd	nd	nd	nd	nd
	Gonad	-	-	-	-	-	-	-	-	-	-	-	-	-	-	-	-	+	+	+	+	nd	nd	nd	nd	nd
	Oviduct	nd	nd	nd	nd	nd	nd	nd	nd	nd	nd	nd	nd	nd	nd	nd	nd	++	-	++	++	nd	nd	nd	nd	nd
	Wattle	nd	nd	nd	nd	nd	nd	nd	nd	nd	nd	nd	nd	nd	nd	nd	nd	+	+	+++	+++	-	-	-	+/-	-

*White, no cells positive; yellow, rare number of cells positive; orange, moderate number cells positive; red many cells positive
"-"=negative, equal to titer <0.5; nd = no data available
**Acinar cells of the pancreas of Eurasian wigeon no. 8 - euthanized at 10 dpi - were +/- postive.
