# Supplementary material for: Wild ducks excrete highly pathogenic avian influenza virus H5N8 (2014–2015) without clinical or pathological evidence of disease
Source: Emerg Microbes Infect. 2018 Apr 18;7:67. doi: 10.1038/s41426-018-0070-9 (PMC5906613; doi:10.1038/s41426-018-0070-9)
Supplement: Supplementary file 7 — Table S4 [file 41426_2018_70_MOESM7_ESM.pdf]

Table S4. Overview of duck species experimentally inoculated with highly pathogenic avian influenza H5 H5 GsGd viruses (part 1 of 2)

Species	Type of duck	Host age	Virus	Subtype	Clade	Culture method	Route of inoculation	Dose of inoculation	Volume (ml)	Clinical signs	Mortality
Mallard	dabbling	adult	A/broiler duck/Kr/Buan2/2014	H5N8	2.3.4.4	egg	IN or CON	10^6.5 EID50	0.1	no or mild	no
Mallard	dabbling	adult	A/chicken/Kr/IS/2006	H5N1	2.2	egg	IN or CON	10^6.5 EID50	0.1	no or mild	no
Mallard	dabbling	adult	A/mandarin duck/PSCC24-24/2010	H5N1	2.3.2.1	egg	IN or CON	10^6.5 EID50	0.1	no or mild	no
Baikal teal	dabbling	adult	A/broiler duck/Kr/Buan2/2014	H5N8	2.3.4.4	egg	IN	10^6.5 EID50	0.1	no	yes
Mallard	dabbling	8-11 mo	A/turkey/Turkey/1/2005	H5N1	2.2.1	egg	IT and IO	10^4 TCID50	3	no	no
Tufted duck	diving	8-11 mo	A/turkey/Turkey/1/2005	H5N1	2.2.1	egg	IT and IO	10^4 TCID50	3	yes	unspecified
Common pochard	diving	8-11 mo	A/turkey/Turkey/1/2005	H5N1	2.2.1	egg	IT and IO	10^4 TCID50	3	yes	yes
Common teal	dabbling	8-11 mo	A/turkey/Turkey/1/2005	H5N1	2.2.1	egg	IT and IO	10^4 TCID50	3	no	no
Eurasian wigeon	dabbling	8-11 mo	A/turkey/Turkey/1/2005	H5N1	2.2.1	egg	IT and IO	10^4 TCID50	3	no	no
Gadwall	dabbling	8-11 mo	A/turkey/Turkey/1/2005	H5N1	2.2.1	egg	IT and IO	10^4 TCID50	3	no	no
Mallard	dabbling	2 wk	A/turkey/Minnesota/12582/2015	H5N2	2.3.4.4	egg in BHI	IN or CON	10^2; 10^4; 10^6 EID50	0.1	no	yes
Mallard	dabbling	2 wk	A/chicken/Iowa/13388/2015	H5N2	2.3.4.4	egg in BHI	IN or CON	10^2; 10^4; 10^6 EID50	0.1	no	no
Mallard	dabbling	2 wk	A/northerin pintail/Washington/40964/2014	H5N2	2.3.4.4	egg in BHI	IN or CON	10^2; 10^4; 10^6 EID50	0.1	no	no
Mallard	dabbling	2 wk	A/gyrfalcon/Washington/40188-6/2014	H5N8	2.3.4.4	egg in BHI	IN or CON	10^2; 10^4; 10^6 EID50	0.1	no	no
Mallard	dabbling	2 wk	A/whooper swan/Mongolia/244/2005	H5N1	2.2	egg in BHI	IN or CON	10^2; 10^4; 10^6 EID50	0.1	yes	yes
Mallard	dabbling	2 wk	A/northerin pintail/Washington/40964/2014	H5N2	2.3.4.4	egg in BHI	IN	10^6 EID50	0.1	yes but 0 dpi not shown	no
Mallard	dabbling	2 wk	A/gyrfalcon/Washington/40188-6/2014	H5N8	2.3.4.4	egg in BHI	IN	10^6 EID50	0.1	yes but 0 dpi not shown	not specified
Mallard	dabbling	2 wk	A/whooper swan/Mongolia/244/2005	H5N1	2.2	egg in BHI	IN	10^6 EID50	0.1	yes	yes
Mallard	dabbling	6 wk	A/goose/Shandong/k1204/2009	H5N5	2.3.4.4	MDCK	IN	10^6 TCID50	0.1	not specified	no
Mallard	dabbling	6 wk	A/goose/Guangdong/k0103/2010	H5N5	2.3.4.4	MDCK	IN	10^6 TCID50	0.1	not specified	no
Mallard	dabbling	6 wk	A/quail/Jiangsu/k0104/2010	H5N5	2.3.4.4	MDCK	IN	10^6 EID50	0.1	yes	no
Mallard	dabbling	6 wk	A/duck/Jiangsu/k1203/2010	H5N8	2.3.4.4	MDCK	IN	10^6 TCID50	0.1	not specified	no
Mandarin duck	dabbling	adult	A/broiler duck/Kr/Buan2/2014	H5N8	2.3.4.4	egg	IN or CON	10^6.5 EID50	0.1	no	no
Mandarin duck	dabbling	adult	A/chicken/Kr/IS/2006	H5N1	2.2	egg	IN or CON	10^6.5 EID50	0.1	no	no
Mandarin duck	dabbling	adult	A/mandarin duck/Kr/PSC24-24/2010	H5N1	2.3.2.1	egg	IN or CON	10^6.5 EID50	0.1	no	no
Redhead	diving	10-16 wk	A/whooper swan/Mongolia/244/2005	H5N1	2.2	egg in BHI	IN	10^6 EID50	0.1	no	no
Wood duck	dabbling	10-16 wk	A/whooper swan/Mongolia/244/2005	H5N1	2.2	egg in BHI	IN	10^6 EID50	0.1	yes	yes
Blue-winged teal	dabbling	10-16 wk	A/whooper swan/Mongolia/244/2005	H5N1	2.2	egg in BHI	IN	10^6 EID50	0.1	no	no
Northern pintail	dabbling	10-16 wk	A/whooper swan/Mongolia/244/2005	H5N1	2.2	egg in BHI	IN	10^6 EID50	0.1	no	no
Mallard	dabbling	10-16 wk	A/duck meat/Anjang/2001	H5N1	0	egg in BHI	IN	10^6 EID50	0.1	no	no
Redhead	diving	10-16 wk	A/duck meat/Anjang/2001	H5N1	0	egg in BHI	IN	10^6 EID50	0.1	no	no
Wood duck	dabbling	10-16 wk	A/duck meat/Anjang/2001	H5N1	0	egg in BHI	IN	10^6 EID50	0.1	yes	yes
Blue-winged teal	dabbling	10-16 wk	A/duck meat/Anjang/2001	H5N1	0	egg in BHI	IN	10^6 EID50	0.1	no	no
Northern pintail	dabbling	10-16 wk	A/duck meat/Anjang/2001	H5N1	0	egg in BHI	IN	10^6 EID50	0.1	no	no
Tufted duck	diving	5 mo	A/duck/Hong Kong/21091/2011	H5N1	2.3.2	MDCK	IT and IO	10^4 TCID50	3	yes	yes

Table S4. Overview of duck species experimentally inoculated with highly pathogenic avian influenza H5 H5 GsGd viruses (part 2 of 2)

Species	Virus excretion Pharynx	Virus excretion Cloacal	Virus excretion Method	Days postinoculation Samples organs	Virus replication (IHC) test done, result int	Virus replication (VI) test done, result int	Reference
Mallard	yes	yes	TCID50/0.1ml per day or tissue	3	no	yes, pos int (pancreas)	Kang et al. 2015
Mallard	yes	no	TCID50/0.1ml per day or tissue	3	no	yes, neg int	Kang et al. 2015
Mallard	yes	no	TCID50/0.1ml per day or tissue	3	no	yes, neg int	Kang et al. 2015
Baikal teal	yes	yes	TCID50/0.1ml per day or tissue	3	no	yes, pos int (pancreas)	Kang et al. 2015
Mallard	yes	no	TCID50/ml and vRNA Ct value per day or tissue; AUC 0-4 dpi	4	yes, neg int	yes, neg int	Keawcharoen et al. 2008
Tufted duck	yes	no	TCID50/ml and vRNA Ct value per day or tissue; AUC 0-4 dpi	4	yes, neg int	yes, pos int	Keawcharoen et al. 2008
Common pochard	yes	yes	TCID50/ml and vRNA Ct value per day or tissue; AUC 0-4 dpi	4	yes, neg int	yes, pos int	Keawcharoen et al. 2008
Common teal	yes	yes	TCID50/ml and vRNA Ct value per day or tissue; AUC 0-4 dpi	4	yes, neg int	yes, neg int	Keawcharoen et al. 2008
Eurasian wigeon	yes	no	TCID50/ml and vRNA Ct value per day or tissue; AUC 0-4 dpi	4	yes, neg int	yes, pos int	Keawcharoen et al. 2008
Gadwall	yes	no	TCID50/ml and vRNA Ct value per day or tissue; AUC 0-4 dpi	4	yes, pos int	yes, pos int	Keawcharoen et al. 2008
Mallard	yes	yes	qPCR used to calculate EID50/ml per day or tissue	3	yes, excl int	no	DeJesus et al. 2016
Mallard	yes	yes	qPCR used to calculate EID50/ml per day or tissue	3	yes, excl int	no	DeJesus et al. 2016
Mallard	yes	yes	qPCR used to calculate EID50/ml per day or tissue	nd	nd	nd	DeJesus et al. 2016
Mallard	yes	yes	qPCR used to calculate EID50/ml per day or tissue	nd	nd	nd	DeJesus et al. 2016
Mallard	yes	yes	qPCR used to calculate EID50/ml per day or tissue	nd	nd	nd	DeJesus et al. 2016
Mallard	yes	yes	qPCR used to calculate EID50/ml per day or tissue	4	yes, neg int	yes, excl int	Pantin-Jackwood et al. 2016
Mallard	yes	yes	qPCR used to calculate EID50/ml per day or tissue	4	yes, neg int	yes, excl int	Pantin-Jackwood et al. 2016
Mallard	yes	yes	qPCR used to calculate EID50/ml per day or tissue	4	yes, pos int (autonomic ganglia enteric tract)	yes, excl int	Pantin-Jackwood et al. 2016
Mallard	yes	yes	TCID50 per day or per gram	5	no	yes, excl int	Zhao et al. 2013
Mallard	yes	yes	TCID50 per day or per gram	5	no	yes, excl int	Zhao et al. 2013
Mallard	yes	yes	EID50 per day or per gram	5	no	yes, excl int	Zhao et al. 2013
Mallard	yes	yes	TCID50 per day or per gram	5	no	yes, excl int	Zhao et al. 2013
Mandarin duck	yes	yes	TCID50/0.1ml per day in figure	3	no	yes, pos int (pancreas) and proventriculus	Kang et al. 2017
Mandarin duck	yes	yes	TCID50/0.1ml per day in figure	3	no	yes, neg int	Kang et al. 2017
Mandarin duck	yes	yes	TCID50/0.1ml per day in figure	3	no	yes, neg int	Kang et al. 2017
Redhead	yes	yes	EID50/ml per tissue; EID50/ml for average max titer; duration (days)	nd	no	yes, neg int	Brown et al. 2006
Wood duck	yes	yes	EID50/ml per tissue; EID50/ml for average max titer; duration (days)	7 and 8	yes, pos int (muscular plexus of small intestines)*	yes, excl int	Brown et al. 2006
Blue-winged teal	yes	yes	EID50/ml per tissue; EID50/ml for average max titer; duration (days)	nd	no	yes, neg int	Brown et al. 2006
Northern pintail	yes	yes	EID50/ml per tissue; EID50/ml for average max titer; duration (days)	nd	no	yes, neg int	Brown et al. 2006
Mallard	yes	yes	EID50/ml per tissue; EID50/ml for average max titer; duration (days)	nd	no	yes, neg int	Brown et al. 2006
Redhead	yes	no	EID50/ml per tissue; EID50/ml for average max titer; duration (days)	nd	no	yes, neg int	Brown et al. 2006
Wood duck	yes	yes	EID50/ml per tissue; EID50/ml for average max titer; duration (days)	not specified	yes, pos int (muscular plexus of small intestines)*	yes, excl int	Brown et al. 2006
Blue-winged teal	yes	no	EID50/ml per tissue; EID50/ml for average max titer; duration (days)	nd	no	yes, neg int	Brown et al. 2006
Northern pintail	yes	no	EID50/ml per tissue; EID50/ml for average max titer; duration (days)	nd	no	yes, neg int	Brown et al. 2006
Tufted duck	yes	yes	TCID50/ml and vRNA Ct value per day or tissue	4	yes, pos int (=pancreas)	yes, pos int (proventriculus, duodenum, pancreas, jejunum, colon)	Bröjer et al. 2015
